# Alpha-CENTAURI: assessing novel centromeric repeat sequence variation with long read sequencing

**DOI:** 10.1093/bioinformatics/btw101

**Published:** 2016-02-24

**Authors:** Volkan Sevim, Ali Bashir, Chen-Shan Chin, Karen H. Miga

**Affiliations:** ^1^Pacific Biosciences, Inc., Menlo Park, CA 94025, USA; ^2^Department of Genetics and Genomic Sciences, Icahn School of Medicine at Mount Sinai, 1425 Madison Avenue, New York, NY 10029, USA; ^3^Icahn Institute for Genomics and Multiscale Biology, Icahn School of Medicine at Mount Sinai, 1425 Madison Avenue, New York, NY 10029, USA; ^4^Center for Biomolecular Science and Engineering, University of California, Santa Cruz, CA 95064, USA

## Abstract

**Motivation:** Long arrays of near-identical tandem repeats are a common feature of centromeric and subtelomeric regions in complex genomes. These sequences present a source of repeat structure diversity that is commonly ignored by standard genomic tools. Unlike reads shorter than the underlying repeat structure that rely on indirect inference methods, e.g. assembly, long reads allow direct inference of satellite higher order repeat structure. To automate characterization of local centromeric tandem repeat sequence variation we have designed Alpha-CENTAURI (ALPHA satellite CENTromeric AUtomated Repeat Identification), that takes advantage of Pacific Bioscience long-reads from whole-genome sequencing datasets. By operating on reads prior to assembly, our approach provides a more comprehensive set of repeat-structure variants and is not impacted by rearrangements or sequence underrepresentation due to misassembly.

**Results:** We demonstrate the utility of Alpha-CENTAURI in characterizing repeat structure for alpha satellite containing reads in the hydatidiform mole (CHM1, haploid-like) genome. The pipeline is designed to report local repeat organization summaries for each read, thereby monitoring rearrangements in repeat units, shifts in repeat orientation and sites of array transition into non-satellite DNA, typically defined by transposable element insertion. We validate the method by showing consistency with existing centromere high order repeat references. Alpha-CENTAURI can, in principle, run on any sequence data, offering a method to generate a sequence repeat resolution that could be readily performed using consensus sequences available for other satellite families in genomes without high-quality reference assemblies.

**Availability and implementation:** Documentation and source code for Alpha-CENTAURI are freely available at http://github.com/volkansevim/alpha-CENTAURI.

**Contact:** ali.bashir@mssm.edu

**Supplementary information:** Supplementary data are available at *Bioinformatics online*.

## 1 Introduction

Centromeres and other heterochromatic regions in most complex genomes are enriched with long arrays of near-identical tandem repeats, known as satellite DNAs, which challenge standard assembly efforts and are vastly underrepresented in genomic studies (Eichler *et al.*, 2004; Willard, 1990). Local arrangements of the fundamental repeat unit, or monomers, within each satellite array are commonly organized into multi-monomeric repeating units or, higher-order repeats (HORs; Willard and Waye, 1987a). These sites are observed to be highly variable in copy number and repeat structure (Warburton and Willard, 1990; Warburton *et al.*, 1993), providing a new and largely uncharacterized source of sequence variation to study in the context of sequence evolution and biomedical research. Previous efforts to characterize repeat variants from whole-genome sequencing (WGS) data relied on predictions made by short read assembly or k-mer frequency (Alkan *et al.*, 2007; Macas *et al.*, 2010). Although useful in predicting the most common repeat unit organizations in a given genome, such strategies are not designed to efficiently survey local rearrangement of repeat patterns involving variant sequence organization between adjacent repeats, precise sites of inversion in arrays and transition boundaries with non-satellite DNA that may interrupt an array. In contrast, long read analysis, where a complete repeating unit can be observed in its entirety more than once, enables direct characterization of these events without assembly based inference (Melters *et al.*, 2013).

To automate the survey of repeat variants in quality-assessed long read datasets, we developed Alpha-CENTAURI (ALPHA satellite CENTromeric AUtomated Repeat Identification), a bioinformatics pipeline for evaluating local HOR structure across long read WGS datasets. When provided with long-read data and a training dataset of repeat units from a satellite family of interest, Alpha-CENTAURI is designed to predict HOR patterns based on clustering discrete repeat units on each read. In doing so, the user is provided with characterization of HOR structure on the read: defined either as *regular*, or containing the same ordering of near-identical monomers, or *irregular*, that is, containing a rearrangement, inversion or discontinuous spacing in monomers when compared to other HORs on the same read. Further, regions of the read that do not contain an identifiable repeat unit are flagged to go through a secondary analysis using Repbase libraries (Jurka *et al.*, 2005) to label sites of characterized repeat insertions. We demonstrate the utility of Alpha-CENTAURI on WGS data from a hydatiform mole genome (CHM1), using a collection of previously characterized 171 bp monomers from an AT-rich human centromeric satellite family, known as alpha satellite (Manuelidis and Wu, 1978).

## 2 Materials and methods

### 2.1 Higher order repeat prediction of ordered monomers

**Fig. 1.**
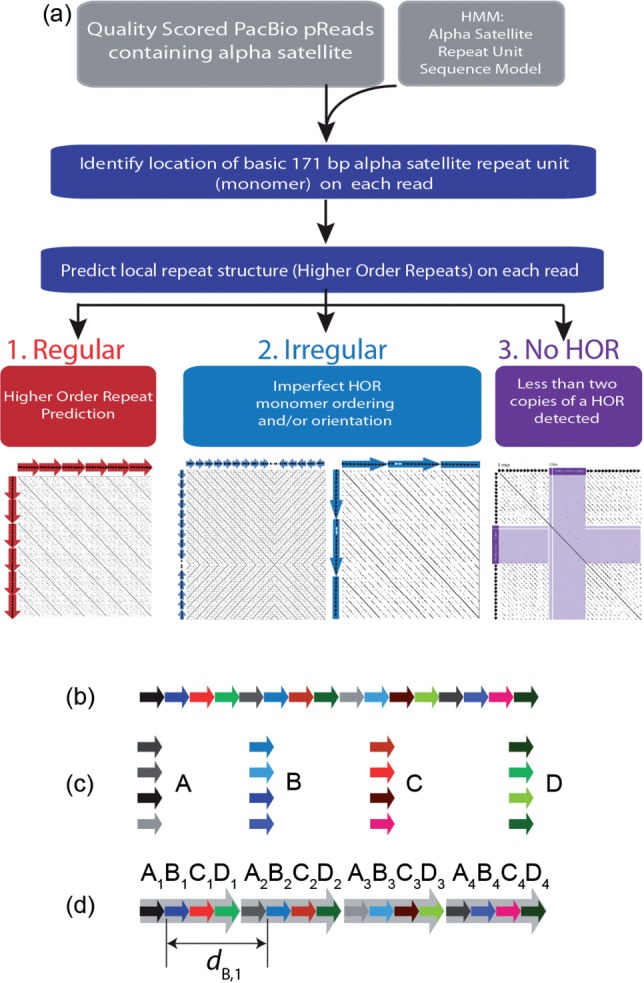
**Alpha-CENTAURI workflow and the HOR detection algorithm illustrated.** (**a**) The workflow. Alpha-CENTAURI takes two input files: a FASTA file containing long reads and an HMM database built using known alpha-satellite monomers. The HMM database is used to infer monomeric sequences in each read. Then, HOR structure is predicted based on the start and end positions of each monomer on the read. The repeat structure on the read is classified under three categories *regular*, *irregular* (including inversion), or cases where no HOR is detected. (**b**) An illustration of a read consisting of an array of alpha-satellite monomers, which are identified from the HMM database. Each block arrow corresponds to a monomer. Similar colors indicate similar sequences. (**c**) Identified monomers are clustered-based sequence similarity. Here, each cluster is labeled by a different letter. (**d**) HOR structure is predicted based on the start positions, end positions and the distances between monomers

Alpha-CENTAURI’s workflow is designed to detect tandem repeats containing at least two ordered monomers (i.e. dimers), providing a minimal definition for HOR prediction (Willard and Waye, 1987b) (Fig. 1). As outlined in Figure 1a, the user is initially required to provide two input databases: (i) a file of quality-assessed long reads and (ii) a monomer training set of fasta sequences to define the basic repeat unit for a given satellite family. Initial monomer positions are determined using a hidden Markov method using a satellite model provided by the input consensus sequence (HMMER, Eddy, 1998). Minimum monomer lengths are defined by default to be 150 bases. Characterization of discrete, ordered monomer units provide an index of start positions for each read. Monomers are clustered into groups based on pair-wise sequence identity using an implementation of an O(*ND*) alignment algorithm (Myers, 1986) within the FALCON genome assembler (https://github.com/PacificBiosciences/FALCON/). Cluster similarity threshold is determined per read by evaluating a range of identity values (98% to 88% by 1% decrements) and selecting the monomer clusters assignments with the highest percent identity that permits inference of HOR organization. Consensus sequence fasta and tab-delimited description of HOR summary statistics are provided for each read.

### 2.2 Evaluation of regular or irregular repeat structures

HOR predictions for each read are classified as *regular* if cluster order, as provided by monomer position-based indexing and similarity clustering, follows a consistent pattern across the entirety of the read. Let, *X*_i_ be the start position of monomer in cluster *X*. Additionally, let *d_X_* be an ordered set of distances between monomers in cluster *X*, where *d_X_*_,i _=_ _*X*_i + 1_-*X*_i_ is the distance between monomer *i* and *i *+* *1 (Fig. 1c), and *d* is the superset of such distances over all clusters. Conditions for regularity are listed below.

1. All identified monomers are assigned to a cluster containing more than one monomer.
2. Distances between the monomers in the same cluster are consistent across the read. Namely, 1.05 ≥ *d_X_*_,i/_Median*(d*) ≥ 0.95.
3. Distance between the tail of each monomer and the head of the adjacent monomer is equal or less than 5 bases.
4. Orientation of all monomers within the same cluster is consistent, i.e. there are no inversions.

Reads that fail any of these conditions are considered *irregular*, while reads failing condition 4 are additionally labeled as inversions. *Irregular* reads contain rearrangements in monomer ordering (regions of monomer insertion and deletion), shifts in orientation of monomer repeat units, and/or stretches of bases that are interspersed or are >5 bp at the start or end of monomers. HORs that contain more than one copy of a certain monomer fail condition 2, and therefore are currently characterized as an irregular. Monomeric satellite structures or highly divergent ordered arrays of monomers that are expected to have single monomer membership in each cluster group, are reported under the no-HOR category.

### 2.3 Evaluation of regular or irregular repeat structures

To evaluate alpha-CENTAURI, we characterized repeat structure predictions using a test dataset of 6438 alpha satellite containing reads (mean read length: 10,536 bp; min: 2000 bp; max: 31,594 bp) from the hydatidiform mole genome (CHM1) (CHM1 error-corrected sequences provided on the Alpha-Centuari git repository.). Predicted repeat structures were compared to a reference database of previously annotated HOR patterns (reviewed Alexandrov *et al.*, 2001) and using monomers obtained from the HuRef genome (Levy *et al.*, 2007; GRCh38 BioProject: PRJNA193213). A summary of repeat regularity is provided in Table 1.

**Table 1.** Centromeric satellite structure predictions

Repeat structure predictions	Reads	Frequency
Regular	1470	0.23
Irregular	3723	0.58
Inversions	154	
No repeat structure detected	1244	0.19

A summary of observed repeat regularity predictions using alpha satellite containing reads from CHM1. This dataset is included with the Alpha-CENTAURI source-code repository.

We observed 23% (1470/6438) of alpha satellite containing reads contained a *regular* HOR. Each *regular* read was assigned to one of 49 previously characterized HORs, with representation across all autosomes and the X chromosome (Supplementary Tables S1 and S2). Within the regular predictions, subsets of reads were observed to describe the same HOR, for example on the X chromosome (DXZ1 or ‘cenX’), 80 reads correctly identified the 12 monomer HOR with a median monomer alignment identity of 98.9%. We were are able to detect HORs that varied in monomer organization within a single array or between nearly identical inter-chromosomal arrays, as seen in the dimeric repeat assigned to chromosome 1, 5 and 19 centromeric regions, where the majority of D1Z7 (or ‘cen1_1’) reads were characterized either as a dimer (66% of reads, 143/218) or a 6-mer repeat (27% of reads, 60/218).

Initially, the majority of predictions were labeled as *irregular*. After alpha satellite sequence annotation, it was determined that a subset of *irregular* assignments were due to missing information: events where HMMER did not correctly predict a monomer position (44 reads, or 1.2% of *irregular* predictions); events where a monomer length was less than the default threshold due to either rearrangement or incomplete HMMER monomer prediction (150 bp); and events where the read length was insufficient to span the full HOR more than once. Additionally, HORs that re-used near-identical monomers present within a single higher order repeat unit, as observed for the D21Z1 HOR, were labeled as *irregular*. The remaining *irregular* reads are expected to represent ‘true’ HOR variants. These include inversions (154, or 4% of *irregular* reads, with the highest prevalence on acrocentric array cen22, an HOR that is found on both chromosomes 14 and 22) as well as rearrangements of the HOR monomer patterns on the same read.

We observed 1244 alpha satellite containing reads that did not provide evidence for an HOR. Roughly 44% (544/1244 reads) were classified a ‘monomeric’ satellites, or highly divergent alpha satellite monomers (i.e. monomers with less than the lower threshold of 88% pairwise identity) typically found in the pericentromeric arms. The remaining 690 reads in our study either represented incomplete HORs single copy HORs or included a transition into non-satellite adjacent sequences (defined here as containing at least 1 kb of non-alpha satellite sequence on the same read). Further analysis of these transitions with RepeatMasker (http://www.repeatmasker.org), using Repbase libraries (Jurka *et al.*, 2005), demonstrates the ability to track sites of transposable element insertion, of which we find LINE1 to be the most prevalent (18.9% of total bases, i.e. 273 042/1 393 472, surveyed from the 140 reads containing at least 1 kb of non-alpha satellite sequence), consistent with the literature of alpha satellite DNA (Schueler *et al.*, 2001).

## 3 Conclusion

We present Alpha-CENTAURI, a python-based workflow for characterizing satellite sequence variants in long read datasets. This automated method is useful in reporting *de novo* higher order repeat sequence organization and predicting structural variation within HORs on a per read basis, thereby characterizing a window of local repeat organization defined by the length of the read. Users are able to rapidly characterize three distinct subgroups of repeat structure, defined here as *regular* or *irregular* higher order repeats, thus defining HOR structures and sites of rearrangement.

While deep, long-read sequencing human datasets are becoming increasingly available, one of the concerns with third-generation sequencing approaches is their relative expensive compared to second-generation technologies at comparable sequence depths. Given the high-copy number of HORs relative to the genome, one can provide highly-informative information of satellite structure at low-depth (potentially even <1× coverage of a genome). Therefore, we believe Alpha-CENTAURI to provide an opportunity to not only provide high-resolution surveys of HORs from deep sequencing projects, but also to enable low depth, low cost surveys across a population or in genomes without high-quality references.

## Funding

This work was supported in part through the computational resources and staff expertise provided by the Department of Scientific Computing at the Icahn School of Medicine at Mount Sinai.

*Conflict of Interest*: C.-S.C. is an employee and a shareholder of Pacific Biosciences, Inc.

## Supplementary Material

Supplementary Data
