# Supplementary material for: Alpha-CENTAURI: assessing novel centromeric repeat sequence variation with long read sequencing
Source: Bioinformatics. 2016 Feb 24;32(13):1921–4. doi: 10.1093/bioinformatics/btw101 (PMC4920115; doi:10.1093/bioinformatics/btw101)
Supplement: Supplementary Data [file supp_32_13_1921__index.html]

Alpha-CENTAURI: assessing novel centromeric repeat sequence variation with long read sequencing — Supplementary Data 

# Alpha-CENTAURI: assessing novel centromeric repeat sequence variation with long read sequencing

## Supplementary Data

files

- Supplementary Data - txt file
- Supplementary Data - pdf file
