# Supplementary material for: Alpha-CENTAURI: assessing novel centromeric repeat sequence variation with long read sequencing
Source: Bioinformatics. 2016 Feb 24;32(13):1921–4. doi: 10.1093/bioinformatics/btw101 (PMC4920115; doi:10.1093/bioinformatics/btw101)
Supplement: Supplementary Data [file supp_btw101_Supplemental_Table_2.pdf]

Supplemental Table 2: Comparison of Alpha-CENTAURI HOR predictions with previously published higher order repeats

**Alpha-CENTAURI Higher Order Repeat Predictions**				**Previously Published Higher Order Repeats**				
**HOR Array ID**	**pReads**	**Predicted HOR (bp)**	**Predicted HOR (Monomer)**	**Locus**	**Chr**	**Published HOR**	**Genbank Accession**	**Reference**
cen1.1	143	340	dimer	D1Z7/D5Z2/D19Z3	1,5,19	2/340	pE25a/M26919-20	Carine et al. (1989);Baldini et al (1989)
cen1.2	5	1866	11-mer	D1Z5	1	11/1900	pSD1-1	Willard and Waye (1978b)
cen2.1	24	1360	8-mer	D2Z1	2	8/1360	M81229	Haaf and Willard (1992)
cen3.1	110	2891	17-mer	D3Z1	3	17/2900	Z12006	Alexandrov et al (1993b)
cen4.1	21	3230	19-mer	D4Z1 (pYAM11-39)	4	20/3200	Z12011-2, p4n1/4, pG-	Mashkova et al (1994)
cen5.1	33	2214	13-mer	D5Z?/D19Z? (pGA16)	5,19	??	pGA16	Hulsebos et al (1988)
cen6.1	51	3057	18-mer	D6Z1	6	20/3222 (or 1	AB005791	Suigmoto et al. (1997);Jabs et al (1984); Jabs and Perisco (1987)
cen7.1	62	1022	6-mer	D7Z1	7	6/1020	M16087, M16101	Waye et al (1987)
cen7.2	5	2734	16mon	D7Z2	7	16/2700	M16037	Waye et al (1987)
cen8.1	54	1868	11mon	D8Z2	8	15/2550	M64779	Ge et al. (1992)
cen10.1	92	1019	6mon	D10Z1	10	6/1020;8/13(	M93288,M93286	Looijenga et al (1992)
cen11.1	127	850	5mon	D11Z1	11	5/850	M21452	Waye et al (1987)
cen12.1	61	1359	8mon	D12Z3	12	8/1395	M93287	Looijenga et al (1992)
cen13.1	4	1870	11mon	D13Z1/D21Z1 (alphaRI,L1.26)	13	11/1868	D29750	Ikeno et al (1994), Greig et al (1993)
cen13.2	1	3914	23mon	D13Z4/D14Z6/D21Z5 (pTRA-2)	13,14,21	??	X55367	Vissel,B. and Choo,K.H. (1991)
cen22.1	132	1364	8mon	D22Z2	22	??	M28299	McDermid et al (1986)
cen15.2-21	9	3415	20mon	D15Z4 (pTRA-25)	15	26/4500	??	Choo et al (1990)
cen16.1	29	1699	10mon	D16Z2	16	10/1700	M58446	Greig et al (1989)
cen17.1	91	2716	16mon	D17Z1	17	16/2712	M13882	Waye and Willard (1986)
cen17.2	20	2379	14mon	D17Z1B	17	14/X	needed	Rudd and Willard (2004)
cen18.1	6	1360	8mon	D18Z1 (pYAM9-60)	18	8/1364	M65181	Alexandrov et al (1991)
cen18.2	24	1701	10mon	D18Z2 (pYAM4-22)	18	10/1700	M65181	Alexandrov et al (1991)
cen20.1	12	2719	16mon	D20Z1 (p3-4)	20	??	??	Waye and Willard (1987)
cen20.2	29	1360	8mon	D20Z2	20	6/1020	X58269	Baldini et al (1992)
cenX.1	80	2057	12-mer	DXZ1	X	12/2000	X02418	Waye and Willard (1985)
